# Metachronous advanced neoplasia after submucosal invasive colorectal cancer resection

**DOI:** 10.1038/s41598-021-81645-2

**Published:** 2021-01-21

**Authors:** Tatsunori Minamide, Hiroaki Ikematsu, Tatsuro Murano, Tomohiro Kadota, Kensuke Shinmura, Yusuke Yoda, Keisuke Hori, Masaaki Ito, Tomonori Yano

**Affiliations:** 1 Department of Gastroenterology and Endoscopy, National Cancer Center Hospital East, 6-5-1, Kashiwanoha, Kashiwa, Chiba 277-8577 Japan; 2 Department of Colorectal Surgery, National Cancer Center Hospital East, 6-5-1, Kashiwanoha, Kashiwa, Chiba 277-8577 Japan

**Keywords:** Cancer, Colonoscopy

## Abstract

Little is known about the incidence of metachronous advanced neoplasia (AN) following resection of submucosal invasive colorectal cancer (SM-CRC). Here, we aimed to assess the occurrence of metachronous AN following SM-CRC resection. We retrospectively reviewed consecutive patients who underwent SM-CRC resection at an academic medical center between 2005 and 2013. Among 343 patients, 250 (72.9%) underwent surgical resection or endoscopic resection followed by surgical resection and 93 (27.1%) underwent only endoscopic resection. During a median follow-up period of 61.5 months, the overall incidence of metachronous AN was 7.6%, and the cumulative incidence at 5 years was 6.1%. The cumulative incidence was significantly higher in the endoscopic resection group than in surgical resection group, in patients with colonic disease than in those with rectal disease, and in patients with synchronous AN than in those without. Multivariate analysis revealed that synchronous AN was the only significant risk factor for metachronous AN (HR 4.35; 95% CI 1.88–10.1). These findings imply that depending on synchronous AN, a surveillance protocol following SM-CRC resection can be changed for better detection of metachronous AN.

## Introduction

Colorectal cancer (CRC) is one of the major causes of cancer-related death worldwide. Surgical resection is the standard treatment for submucosal invasive CRC (SM-CRC), because lymph node metastases are detected in approximately 6–12% patients^1–5^. However, endoscopic resection is acceptable for select cases of SM-CRC, considering the low incidence of lymph node metastasis. According to the Paris classification and the Japanese Society for Cancer of the Colon and Rectum (JSCCR) guidelines, patients with SM-CRC who have any of the following histopathological characteristics are considered to be at high-risk for lymph node metastasis: (i) positive vertical margin, (ii) depth of submucosal invasion > 1000 μm, (iii) lymphovascular invasion, (iv) poorly differentiated adenocarcinoma, and (v) budding grade of BD2/3^6–8^. Conversely, patients with SM-CRC without these factors are regarded to be at low-risk for lymph node metastasis. Depending on this risk classification, surgical resection is recommended for high-risk SM-CRC, whereas endoscopic resection alone is adequate for low-risk SM-CRC. The long-term outcomes of this therapeutic selection have been reported in patients with SM-CRC^9,10^.

Surveillance colonoscopy after SM-CRC resection is recommended for detection of local recurrence or residual tumor and detection of metachronous colorectal neoplasia (CRN), particularly advanced neoplasia (AN). AN includes invasive cancer and advanced adenoma, with the latter being related to an increased risk for subsequent CRC^11–13^. Based on studies reporting a high frequency of metachronous CRC, the current guidelines recommend surveillance colonoscopy at least 1 year following surgical resection of CRC^8,14–17^. Conversely, surveillance colonoscopy is recommended 3 years after endoscopic resection of AN to manage the risk of metachronous AN^18,19^. However, there ls little evidence for the incidence of metachronous AN after surgical and endoscopic resection of SM-CRC. Therefore, this study aimed at investigating the metachronous AN incidence following SM-CRC resection.

## Methods

### Study population

We retrospectively reviewed consecutive patients at the National Cancer Center Hospital East, Kashiwa, Japan, who were treated by surgical or endoscopic resection for SM-CRC between 2005 and 2013. Inclusion criteria were as follows: (i) histologically proven complete resection of SM-CRC, (ii) resection of every other CRNs before SM-CRC resection including any small adenomas before SM-CRC resection, and (iii) one or more total surveillance colonoscopies following SM-CRC resection. We excluded patients with (i) inflammatory bowel disease, familial adenomatous polyposis syndrome, Lynch syndrome (diagnosed by germline genetic testing after reviewing personal and family histories), or synchronous advanced CRC; (ii) a history of surgical colorectal resection or preoperative/postoperative chemotherapy; and (iii) a follow-up period of < 1 year.

The study was approved by the institutional review board (registration number 2018-067, approval date 06/18/2018) and performed according to the ethical principles outlined in the Declaration of Helsinki. All participants gave written informed consent for examination and treatment prior to procedures.

### Data collection

We obtained the following data from the electronic medical records: age, sex, lesion characteristics of all CRN, including SM-CRC (location, size, morphology, and histopathological diagnosis), resection method, and follow-up data. The SM-CRC resection methods were classified into 2 groups: surgical resection only or endoscopic resection followed by surgical resection (SR group) and endoscopic resection only (ER group). Follow-up information included the date of surveillance total colonoscopy, presence of residual tumor or local recurrence of SM-CRC after resection, and characteristics of metachronous AN lesion. The information from surveillance colonoscopy within 6 months after pre-resection colonoscopy for SM-CRC was included in that from pre-resection colonoscopy to decrease the number of missed lesions.

### Endoscopic procedure

For bowel preparation, all patients were orally administered 1–2 L of hypertonic polyethylene glycol solution or 1.8 L of magnesium citrate. Scopolamine butylbromide or glucagon was administered to inhibit bowel peristalsis, and pethidine hydrochloride and/or midazolam were used for conscious sedation.

Magnifying colonoscopes were used for this study (PCF-Q240ZI, PCF-Q260ZI, and PCF-H290ZI; Olympus, Tokyo, Japan). Detected lesions were examined by narrow-band imaging and/or chromoendoscopy including 0.4% indigo carmine dye and 0.05% crystal violet in conjunction with the magnifying function. Lesions with a non-invasive pattern were diagnosed as adenoma, intramucosal CRC (high-grade dysplasia), or superficial SM-CRC, and resected endoscopically^20–22^. Lesions with an invasive pattern were diagnosed as deep SM-CRC and recommended for surgical resection; however, they were resected endoscopically only if the patients refused surgical resection.

Endoscopic resection included endoscopic submucosal dissection (ESD), endoscopic mucosal resection (EMR), endoscopic piecemeal mucosal resection (EPMR), and polypectomy. Transanal local excision was also considered as endoscopic resection as it was non-curative.

All participating endoscopists had experienced at least 200 cases of colonoscopic procedures.

### Surgical procedure

The lesions evaluated as deep SM-CRC were curatively resected including lymph node dissection, with the patient’s consent. If the histopathological findings of endoscopically resected SM-CRC revealed any of the risk factors proposed by the JSCCR guidelines, additional surgical resection with lymph node dissection was recommended.

### Histopathological examination

Formalin-fixed specimens were stained with hematoxylin and eosin. The histopathological diagnosis was determined according to the World Health Organization classification and JSCCR guidelines^8,23^. We classified SM-CRC cases with positive vertical margins, depth of submucosal invasion > 1000 μm, lymphovascular invasion, poorly differentiated adenocarcinoma, and budding grade of BD2/3 into a high-risk group for lymph node metastasis^6–8^. SM-CRC cases without these factors were classified into the low-risk group.

### Follow-up

Surveillance total colonoscopies were performed at least 1 and 5 years after SM-CRC resection, although the attending physicians decided the precise surveillance interval and period for colonoscopies. Any newly detected CRNs including small adenomas were resected during surveillance colonoscopies. Blood tests, chest radiography, and computed tomography were also performed for the detection of local and distant recurrences over 5 years.

### Outcomes

The study outcomes were the overall and cumulative incidence rates of metachronous AN detected from surveillance colonoscopies. Characteristics of metachronous AN and risk factors for metachronous AN incidence were also analyzed. AN was defined as adenoma ≥ 10 mm, adenoma with villous histology, adenoma with high-grade dysplasia, or invasive cancer. Metachronous AN was defined as AN detected at least 6 months after pre-resection colonoscopy for SM-CRC. Synchronous AN was defined as AN detected in pre-resection or surveillance colonoscopy within 6 months after pre-resection colonoscopy.

### Statistics

Categorical variables are expressed as frequencies (%) and were compared using Fisher’s exact test. Continuous variables are expressed as medians with interquartile ranges (IQRs) and were analyzed using the Mann–Whitney *U* test. The Kaplan–Meier method was used for calculation of the cumulative incidence of metachronous AN, and the log-rank test was used to compare groups. A Cox proportional hazards model was applied to evaluate the hazard ratio (HR) and 95% confidence interval (CI) for metachronous AN incidence after adjusting for potential confounders. The follow-up period was defined from the day of the total colonoscopy before SM-CRC resection to the last surveillance total colonoscopy. If local or distant recurrences were detected by imaging, the end of the follow-up period was defined as the date of the last surveillance total colonoscopy before detection. All tests were 2-tailed, and a *P* value of < 0.05 was considered statistically significant. All statistical tests were conducted using EZR (Saitama Medical Center, Jichi Medical University, Saitama, Japan), a graphical user interface for R (The R Foundation for Statistical Computing, Vienna, Austria).

## Results

### Baseline characteristics

**Figure 1:**
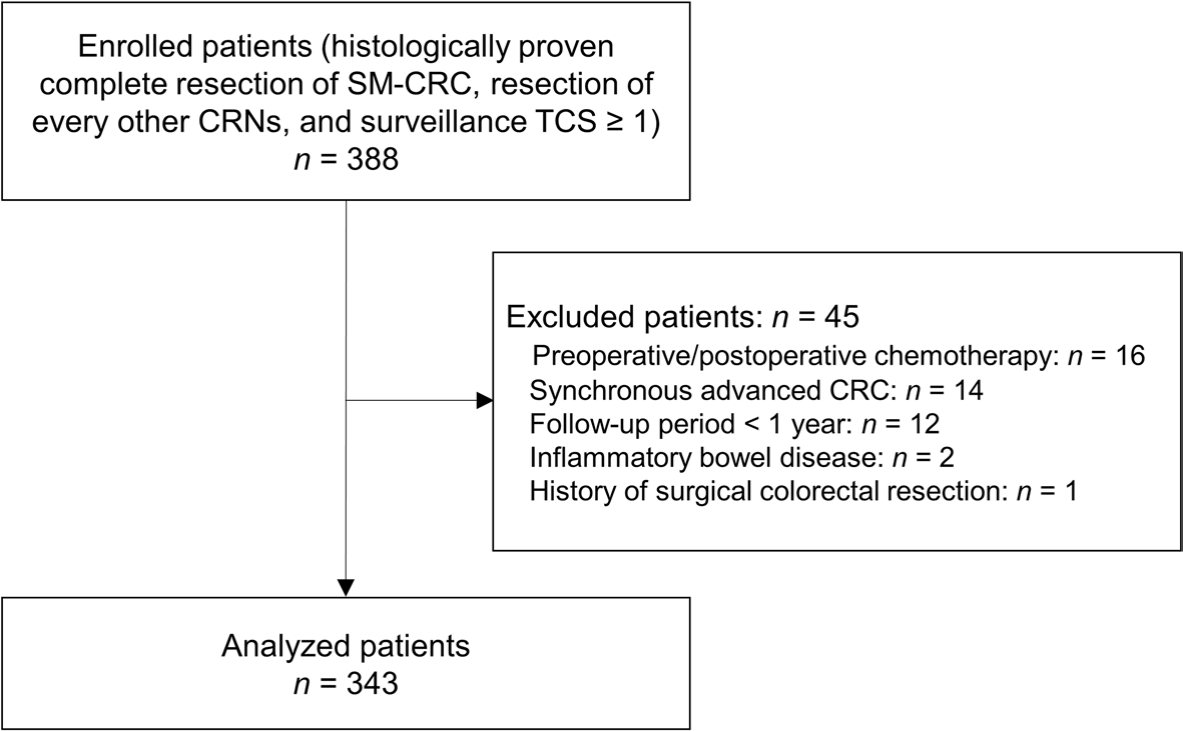
Flowchart of the study population. *SM-CRC* submucosal invasive colorectal cancer, *CRN* colorectal neoplasia, *TCS* total colonoscopy.

A total of 388 consecutive patients were enrolled according to the inclusion criteria. After excluding 45 patients, we eventually analyzed 343 patients (Fig. 1). The baseline characteristics of patients and SM-CRCs in the study population are summarized in Table 1. In the ER group, ESD, EMR, EPMR, polypectomy, and transanal local excision were performed in 48, 25, 7, 7, and 6 patients, respectively. None of the patients had residual tumors. Nevertheless, 5 patients (4 in the SR group and 1 in the ER group) experienced local or distant recurrence after SM-CRC resection (median period until recurrence: 61.9 months; range 14.7–63.4 months).

**Table 1:** Baseline characteristics of patients and submucosal invasive colorectal cancer.

	Number of patients (*n* = 343)	%
Age, median (IQR), years	65 (59–71)	
**Sex**		
Male	219	63.8
Female	124	36.2
**Lesion location**		
Right colon	100	29.2
Left colon	131	38.2
Rectum	112	32.7
**Treatment**		
Surgical resection only	145	42.3
Endoscopic and additional surgical resection	105	30.6
Endoscopic resection only	93	27.1
**Histopathological risk**		
Low	61	17.8
High	280	81.6
Unknown	2	0.6
**Synchronous advanced neoplasia**		
No	254	74.1
Yes	89	25.9
Number of surveillance total colonoscopies, median (IQR)	2 (2–4)	
Follow-up period, median (IQR), months	61.5 (41.4–66.2)	

*IQR* interquartile range.

### Overall incidence of metachronous AN

The overall incidence rate of metachronous AN detected through surveillance colonoscopies after SM-CRC resection was 7.6% (26/343 patients; 95% CI 0.5–10.9%). This outcome was significantly more frequent in the ER group (17.2%; 16/93 patients) than in the SR group (4.0%; 10/250 patients, *P* < 0.001), and in patients with colonic SM-CRC (colon group, 10.0%; 23/231 patients) than in those with rectal SM-CRC (rectal group, 2.7%; 3/112 patients, *P* = 0.016). The overall incidence rate of metachronous AN was also significantly higher in patients with synchronous AN (18.0%; 16/89 patients) than that in those without synchronous AN (3.9%; 10/254 patients, *P* < 0.001).

### Cumulative incidence of metachronous AN

**Figure 2:**
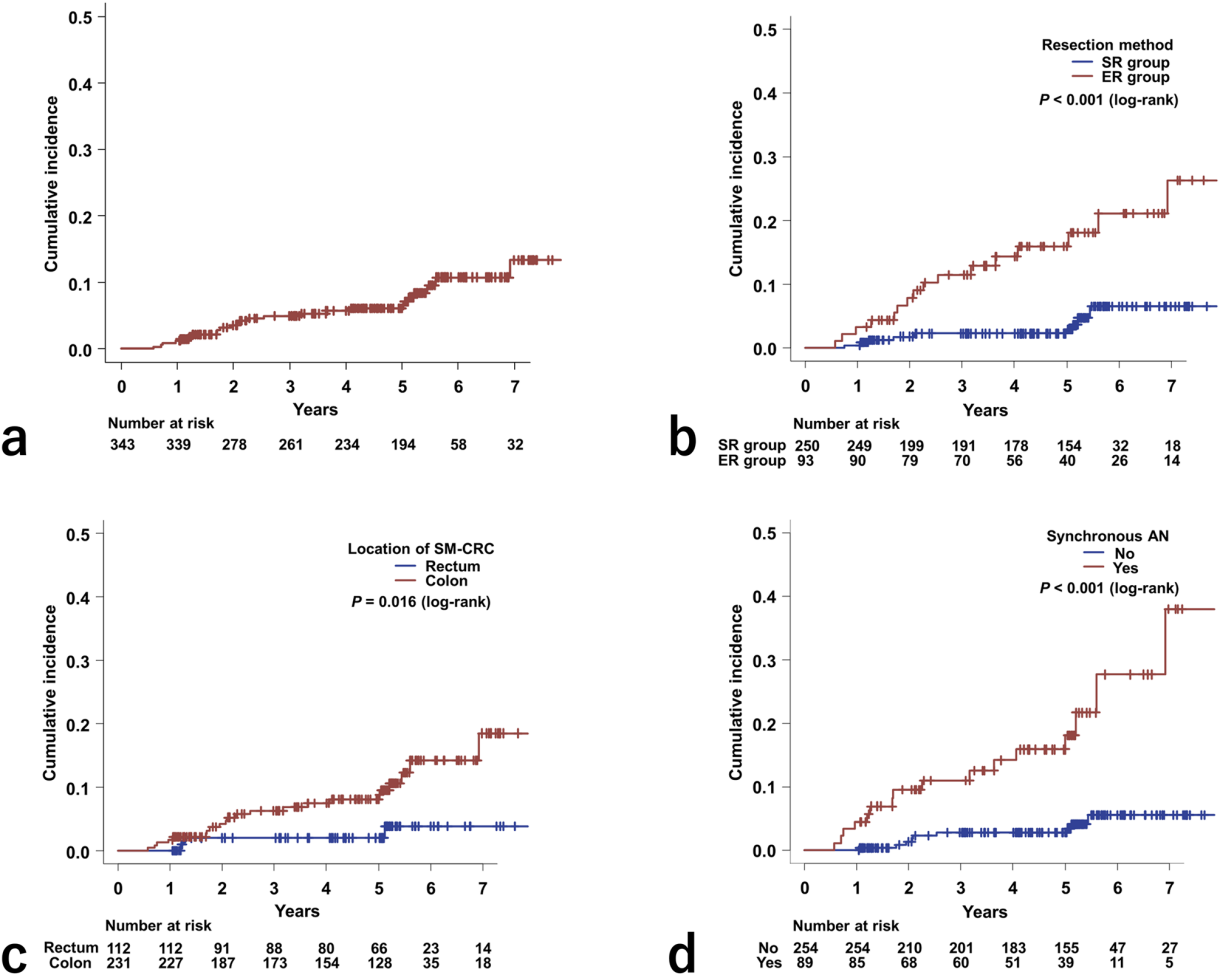
Cumulative incidence of metachronous advanced neoplasia after resection of submucosal invasive colorectal cancer (**a**) in the entire study group, (**b**) according to the resection method, (**c**) according to the location of submucosal invasive colorectal cancer, and (**d**) according to the presence of synchronous advanced neoplasia. *ER* endoscopic resection, *SR* surgical resection, *SM-CRC* submucosal invasive colorectal cancer, *AN* advanced neoplasia.

Figure 2a shows the overall cumulative incidence of metachronous AN following SM-CRC resection. The 5-year overall cumulative incidence rate was 6.1%.

Figure 2b shows the cumulative incidence of metachronous AN according to SM-CRC resection method. This outcome was significantly more frequent in the ER group than in the SR group (*P* < 0.001). The 5-year cumulative incidence rates in the ER and SR groups were 15.9% and 2.2%, respectively.

Figure 2c shows the cumulative incidence of metachronous AN according to SM-CRC location. The colon group showed a significantly higher cumulative incidence rate than did the rectal group (*P* = 0.016). In the colon group, the cumulative incidence rate at 5 years was 8.1%; this was only 2.0% in the rectal group.

Figure 2d shows the cumulative incidence of metachronous AN depending on the presence of synchronous AN. This outcome was significantly more frequent in the group with synchronous AN than that in the group without synchronous AN (*P* < 0.001). In the group with synchronous AN, the cumulative incidence rate at 5 years was 15.8%. For the group without synchronous AN, the cumulative incidence rate at 5 years was 2.8%.

### Metachronous AN characteristics

In total, 32 metachronous ANs were detected in 26 patients after SM-CRC resection (Table 2). Using the Aronchick scale, bowel preparation in the most recent colonoscopy was poor in only 3 cases (9.4%)^24^. More than half of metachronous ANs were found in the ER group, in the right colon and nonpolypoid. The median period from pre-resection colonoscopy to detection of metachronous AN was 33.8 months.

**Table 2:** Clinicopathological characteristics of metachronous advanced neoplasia after resection of submucosal invasive colorectal cancer.

Characteristics	Number of patients (*n* = 32)	%
**Bowel preparation in the most recent colonoscopy**		
Excellent	10	31.3
Good	15	46.9
Fair	4	12.5
Poor	3	9.4
**Resection method for SM-CRC**		
SR group	13	40.6
ER group	19	59.4
**Lesion location**		
Right colon	17	53.1
Left colon	11	34.4
Rectum	4	12.5
Size, median (IQR), mm	12 (10–15.3)	
**Morphology**		
Nonpolypoid	22	68.8
Polypoid	10	31.3
**Histopathology**		
Tubular adenoma ≥ 10 mm	22	68.8
High-grade dysplasia	12	37.5
Invasive cancer	5	15.6
Adenoma with villous histology	0	0
Period from pre-resection colonoscopy to detection, median (IQR), months	33.8 (20.5–63.2)	

*ER* endoscopic resection, *IQR* interquartile range, *SM-CRC* submucosal invasive colorectal cancer, *SR* surgical resection.

Among the 5 metachronous invasive cancers detected on surveillance colonoscopies, 3 were SM-CRC, and 2 were advanced CRC. Four invasive cancers, including 2 advanced CRCs, were found in the SR group. The median period from pre-resection colonoscopy to detection of invasive cancers was 48.8 months (range 14.6–122.3 months).

### Risk factors for metachronous AN

A Cox proportional hazards model was used to analyze the risk factors associated with metachronous AN following SM-CRC resection (Table 3). This multivariate analysis demonstrated a significant correlation between synchronous AN and the risk of metachronous AN (HR 4.35; 95% CI 1.88–10.1), after adjustment for age, sex, location (colon or rectum), resection method (SR or ER), and number of surveillance colonoscopies (< 3 or ≥ 3).

**Table 3:** Multivariate analysis of risk factors for metachronous advanced neoplasia after resection of submucosal invasive colorectal cancer.

	Number of patients (*n* = 343)	Metachronous advanced neoplasia, *n*	HR (95% CI)	*P* value
**Age**				0.156
< 65 years	169	8	1	
≥ 65 years	174	18	1.88 (0.79–4.47)	
**Sex**				0.200
Female	124	3	1	
Male	219	23	2.27 (0.65–7.94)	
**Lesion location**				0.213
Rectum	112	3	1	
Colon	231	23	2.25 (0.63–8.09)	
**Resection method**				0.054
SR group	250	10	1	
ER group	93	16	2.34 (0.98–5.57)	
**Synchronous advanced neoplasia**				< 0.001
No	254	10	1	
Yes	89	16	4.35 (1.88–10.1)	
**Number of surveillance total colonoscopy**				0.062
< 3 times	178	3	1	
≥ 3 times	165	23	3.45 (0.94–12.6)	

*CI* confidence interval, *HR* hazard ratio, *SR* surgical resection, *ER* endoscopic resection.

## Discussion

The present study assessed the metachronous AN incidence following SM-CRC resection over a median follow-up period of 5 years. We compared the incidence of metachronous AN according to clinicopathological factors, and found that endoscopic resection, colonic SM-CRC, and synchronous AN were significantly related to high overall and cumulative incidence rates. Moreover, we found an independent association between synchronous AN and the risk of metachronous AN after SM-CRC resection.

We set the outcomes as occurrence of metachronous AN following SM-CRC resection and associated factors, as AN is considered to represent the optimal target lesion for CRC screening^11,25–27^. Adenoma characteristics and metachronous AN were significantly associated^11,12^. The United States Multi-Society Task Force (USMSTF) and the European Society of Gastrointestinal Endoscopy recommend surveillance colonoscopy at 3 years post resection for high-risk patients (adenoma ≥ 10 mm, adenoma with villous histology, adenoma with high-grade dysplasia, or ≥ 3 adenomas)^18,19^. A Korean prospective study showed that the 5-year cumulative incidence rate of metachronous AN was 12.2% in high-risk patients^28^, whereas, in a Japanese retrospective study, the 5-year cumulative incidence rate of metachronous AN was 12.6% in patients with intramucosal cancer (high-grade dysplasia) on baseline colonoscopy^29^. In our study, the cumulative incidence rate at 5 years after endoscopic resection of SM-CRC was 15.9%. These results suggest the necessity of surveillance after endoscopic resection of SM-CRC, considering the high risk for metachronous AN.

In contrast, surveillance colonoscopy is recommended at least 1 year following surgical resection of CRC by the USMSTF, European Society for Medical Oncology, and JSCCR guidelines^8,16,17^, based on the high cumulative incidence rate of metachronous CRC within few years after the initial diagnosis, with an estimated annual incidence rate of 0.3–0.35% after surgical resection^15,16^. A Dutch retrospective population-based study reported that 93 patients (1.8%) developed metachronous CRC, with an 81-month mean interval between the diagnoses of primary and metachronous lesions^14^. In the present study, 4 patients (1.6%) were diagnosed with metachronous CRC during follow-up after surgical resection of SM-CRC. This result is consistent with that of previous large-scale studies, which included patients with more advanced CRC and might indicate that patients with SM-CRC also have a risk for metachronous CRC.

The ER group were at significantly higher risk for metachronous AN than the SR group. Notably, the 5-year cumulative incidence rate was much higher in the ER group than in the SR group (15.9% vs. 2.2%). This result could be partly attributable to the differences in the length of the residual intestinal tract. However, there was more than a twofold difference in the cumulative incidence between the 2 groups, which is difficult to explain based on a remnant length difference. Another explanation is that the located segment served as an environmental risk factor for SM-CRC, as well as for metachronous AN. The location of metachronous AN was similar to that of SM-CRC in 6 lesions and adjacent to SM-CRC in 4 of 19 lesions detected in the ER group. Although incomplete endoscopic removal of SM-CRC can cause a residual/recurrent lesion in the same segment, mimicking metachronous AN, complete resection was histopathologically proven in all enrolled cases. These findings suggest that the location of SM-CRC might be associated with the occurrence of metachronous AN, which is supported by recent studies that revealed molecular differences according to location in the colorectum. This shows the possibility of varying, location-dependent carcinogenic risks^30,31^.

Multivariate analyses demonstrated that synchronous AN was significantly associated with the risk of metachronous AN after SM-CRC resection (HR 4.35; 95% CI 1.88–10.1). Synchronous CRC was reported to be one of the risk factors related to the occurrence of metachronous CRC after surgical resection^15,32–34^. A Japanese retrospective study revealed a correlation between synchronous AN and a higher risk of metachronous AN following surgical resection of CRC^35^. Although we included endoscopically resected SM-CRC, the present findings are similar to those in previous reports. Therefore, a surveillance protocol after SM-CRC resection can be modified depending on synchronous AN. However, a larger prospective research is required to explore this point especially after endoscopic resection of SM-CRC.

The strength of our study is the inclusion of patients after surgical and endoscopic resection of SM-CRC with long follow-up periods. Although local recurrence following endoscopic resection of SM-CRC was previously investigated^9^, we believe that the present study is the first to demonstrate the incidence of metachronous AN following endoscopic resection of SM-CRC. However, there are several limitations. First, this was a retrospective single-center study conducted at an academic medical hospital. Hence, the present findings may not be generalizable to a broader population. However, the present study had an overall cumulative incidence of metachronous CRC of 1.6% following surgical resection of SM-CRC, which is similar to that of a previous large-scale study^14^. Second, the surveillance schedule was not identical among patients because of the retrospective design of the study. To overcome this limitation, we applied the Kaplan–Meier method for calculation of the cumulative incidence of metachronous AN. Moreover, we included the number of surveillance colonoscopies in the multivariate analysis of risk factors for metachronous AN. Third, the sessile serrated lesions (SSLs) were resected according to the endoscopist’s decision, without any established strategy. However, SSLs were not noted before the detection of metachronous ANs, even though more than half of metachronous ANs were nonpolypoid and of the right colon. Fourth, we could not obtain detailed information regarding the lifestyle factors and endoscopists’ accuracy of lesion detection. Therefore, there may be confounding variables that were omitted from the multivariate analysis. Finally, missed lesions might contribute to the metachronous AN incidence. One population-based study found that, in most cases, metachronous CRC after CRC resection could be explained by missed lesions^14^. This previous study considered metachronous CRC diagnosed within 36 months after the last colonoscopy as a missed lesion. All 5 CRC cases detected on surveillance colonoscopies fit this definition. Therefore, our findings might not reflect the exact metachronous AN incidence.

In conclusion, the current study showed that synchronous AN was significantly related to a high incidence of metachronous AN following SM-CRC resection. For better detection of metachronous AN, a surveillance protocol following SM-CRC resection can be changed depending on synchronous AN.

## Data Availability

The datasets generated during and/or analyzed during the current study are available from the corresponding author on reasonable request.
